# Long-term use of metformin and colorectal cancer risk in type II diabetics: a population-based case–control study

**DOI:** 10.1002/cam4.306

**Published:** 2014-08-05

**Authors:** Majken Cardel, Sara M Jensen, Anton Pottegård, Trine L Jørgensen, Jesper Hallas

**Affiliations:** 1 Hospital Pharmacy, Hospital Lillebaelt, VejleRegion South, Denmark; 2 Nordisk Center for Jordens Udvikling (NordCEE), University of Southern DenmarkOdense, Denmark; 3 Research Unit of Clinical Pharmacology, Institute of Public Health, University of Southern DenmarkOdense, Denmark; 4 Department of Oncology, Odense University HospitalOdense, Denmark

**Keywords:** Cancer prevention, colorectal cancer, metformin, population-based case–control study, type II diabetes mellitus

## Abstract

In vitro and animal studies indicate that metformin prevents colorectal cancer (CRC). Epidemiological studies, however, have been equivocal. We undertook this study to assess whether metformin prevents CRC in individuals with type II diabetes. We performed a nested case–control study restricted to Danish citizens with type II diabetes. Data were collected from four Danish nationwide registries. Cases were type II diabetics with a primary CRC between 2000 and 2009, and controls were sampled among subjects with type II diabetes. Long-term exposure to metformin was defined by the redeeming of prescriptions for a cumulative dose of 2000 g within 5 years prior to the index date. To control for potential confounders, we used unconditional logistic regression. We generated adjusted odds ratios (OR) for the association between metformin and CRC and performed subanalyses for selected subgroups and for the dose–response relation. We identified 2088 cases and 9060 controls during the study period. The association between long-term metformin use and CRC gave an adjusted OR at 0.83 (95% CI 0.68–1.00). A protective effect on CRC with long-term use of metformin was only evident for women (OR 0.66 vs. 0.99 for men). There was a significant dose–response association of metformin use >250 defined daily dose (DDD) and for the duration of metformin use >1 year. We found an indication of a protective effect of long-term metformin use against CRC in type II diabetics, although this effect was only seen in women.

## Introduction

Metformin is a widely used antidiabetic agent with over 120 million users worldwide in 2010 1. It is the first-line glucose-lowering therapy used in the treatment of type II diabetes in conjunction with lifestyle changes 2. Several experimental studies have shown that metformin is likely to have anticancerous effects in both in vivo and in vitro settings 3–5. Studies in rodents have shown that metformin is able to slow tumor progression and growth and to reduce the number of aberrant crypt foci (ACF) through the activation of 5′AMP-activated kinase (AMPK) 6,7. The activation of AMPK leads to several cellular events such as inhibition of cell proliferation, angiogenesis, and fatty acid synthesis and induction of cell cycle arrest, autophagy, and apoptosis 8–14. Unrelated to its antidiabetic properties, epidemiological studies have shown promising results toward a protective effect of metformin on colorectal cancer (CRC), although not consistently 3–5,15. Because of the conflicting epidemiologic evidence, we conducted a nested case–control study with the aim of determining whether long-term use of metformin has a preventive effect against CRC among type II diabetics. Since CRC is the third most common type of cancer and has a high mortality rate, a possible antitumor effect of metformin would have considerable public health impact 16.

## Subjects and Methods

The study was conducted as a population-based case–control study of incident CRCs in Danish citizens during the period of 1 January 2000 to 31 December 2009. Type II diabetes is the main indication for metformin use and is by itself a risk factor for CRC. Thus, to minimize confounding by indication, we restricted the study population to subjects with a diagnosis of type II diabetes.

### Data sources

Four nationwide registers were used to generate the data: the Danish Cancer Registry (DCR), the Danish National Patient Register (NPR), the Danish National Prescription Registry (DNPR), and the Danish Civil Registration System (CRS).

Cases were identified by the DCR 17,18, which has recorded incident cases of cancer on a nationwide basis since 1943. The DCR contains accurate and specific information about tumors characterized by the International Classification of Diseases (ICD) for oncology ICD-O-3 from 1977 to 2003 and ICD-10 codes thereafter. Since reporting of cancers to the DCR is mandatory, the registry is almost complete.

Since 1977 all hospitalizations in Denmark have been recorded in the NPR 19. The diagnoses were encoded according to ICD-8 from 1978 to 1993, and from 1994 ICD-10. Since the National Health Board offers universal coverage, free of charge for all Danish citizens, the DNPR allows true population-based studies of disease occurrence. Diagnoses for both in-patient and out-patient contacts are recorded.

We retrieved data on the drug use of the study objects from the DNPR. DNPR has recorded information on all redeemed prescription drugs on individual user level since 1994 20. The recorded data include the prescription holder, the date of dispensing, the substance, quantity among other variables. Drugs are categorized according to the Anatomic Therapeutic Chemical (ATC) code, a hierarchical classification system developed by the World Health Organization (WHO) for purposes of drug use statistics 20. We used the defined daily dose (DDD) to express the drug quantity dispensed for each prescription 20.The DDD of a drug, also established by WHO, is its assumed average adult maintenance dose when administered for its main indication as mono-therapy 20.

The CRS 21 was used to retrieve information of the cases' and controls’ vital status (date of death) and migration or emigration, which allowed us to keep track of the eligibility of subjects as controls and to ensure that all subjects had a complete history of drug use and hospital care contacts.

All Danish citizens have a unique personal identification number, which linked all data sources together 21. Statistics Denmark, a governmental institution, performed the linkage of data 20.

### Cases

Cases were all Danish type II diabetic patients (ICD-8 250.00 diabetes mellitus, insulino independente, sine complicatione, and /or ICD-10 DE11) with a primary CRC diagnosis (ICD-7: 1530–1535, 4530–4538, 8530–8535, 2530–2534; ICD-10: DC18, DC18.0–18.9 or ICD-7: 1540; ICD-10 DC20) between 1 January 2000 and 31 December 2009 and who did not fulfill one of the exclusion criteria. We only included cases with histological confirmation of the cancer diagnosis. Cases were excluded if they were not inhabitants in Denmark at the date of the cancer diagnosis (index date), migrated to or from Denmark less than 10 years before the index date, or if they were younger than 40 years of age. If cases had any of the following diagnoses, they were also excluded: Polycystic Ovarian Syndrome (PCOS) (ICD-8: 256.9; ICD-10: DE282) or Inflammatory Bowel Disease (IBD) (ICD-8: 563.01, 563.19, 569.04; ICD-10: DK50.0-50.9, DK51.0-51.9). Finally, we excluded subjects who had a type I diabetes diagnosis before their type II diabetes diagnosis. The purpose was to prevent the wrongful inclusion of patients with type I diabetes that were mistakenly coded with type II diabetes at one time in their medical record.

### Controls

Controls were selected by incidence density sampling. In brief, we established a random sample of all type II diabetics in Denmark and assigned each subject a random index date between 1 January 2000 and 31 December 2009. The exclusion criteria for cases were also applied to controls. The initial number of controls was 10,000, thus aiming at a control:case ratio of 4:1. As some of the exclusion criteria were applied after controls selection procedure, the final control: case ratio deviated slightly from 4:1. Study subjects were eligible to be selected as controls before they became cases. Thereby, the computed odds ratio (OR) is an unbiased estimate of the incidence rate ratio that would have emerged from a cohort study of the same source population 22.

### Exposure definition

Cases and controls were considered ever-users of metformin (ATC: A10BA02, A10BD03, A10BD07, A10BD08) if they had redeemed at least one prescription for metformin or combination medicine with metformin prior to the index date. Study subjects were considered long-term users if they had been exposed to metformin for a cumulative dose of 1000 DDD within 5 years prior to the index date. WHO has defined the DDD of Metformin as 2 g 23. Combination drugs containing metformin were included in the exposure definition by using data on the metformin component of the drug and then applying the DDD value for metformin.

In order to describe the duration–response association between metformin use and CRC risk, we calculated the cumulative duration of metformin use for each subject. Since metformin may be used episodically, this calculation entailed an assessment of which prescriptions belonged to the same treatment episode 24. To define the exposure duration that should be assigned to each prescription, we performed an analysis of waiting time distribution (WTD) 25. The WTD analysis showed that if there was more than 11 weeks between redeeming of two metformin prescriptions, they were unlikely to belong to the same treatment episode. Thus we assigned each metformin-prescription an exposure period of 11 weeks, that is, 77 days. If a gap of more than 77 days was observed between two prescriptions it was assumed that the drug had been paused or seponated 24.

### Analysis

The study was an unmatched case–control study. ORs for cancer risk associated with metformin exposure were calculated using unconditional logistic regression with adjustment for potential confounders including age and gender. In all analyses the use of metformin was analyzed with never-use of metformin as reference.

The following potential confounders were included in the regression model: a) Use of drugs known or suspected to modify the risk of CRCs including aspirin (ATC: B01AC06, N02BA01, N02BA51, B01AC30), nonaspirin NSAIDs excluding glukosamine (ATC: M01A; exclude ATC: M01AX05), Statins (ATC: C10AA), SU-drugs such as Glibenclamide (ATC: A10BB01) and Gliclazide (ATC: A10BB09), Rosiglitazone (A10BD03) or Insulin glargine (A10AE04). Exposure to a confounder drug was defined by a cumulative dose of at least 500 DDD prior to the index date except for aspirin as it was defined as redemption of at least 400 tablets within 5 years prior to index date. This was determined by an analysis of prescription patterns of the Danish citizens by MEDSTAT 26. b) Life style confounders, which could potentially modify the CRC risk. These were a diagnosis of obesity (iCD-8: 277.99; ICD-10: DE66), chronic obstructive pulmonary disease (COPD) (as a crude marker of heavy smoking) (ICD-8: 490.00,491.00, 491.01,491.03; ICD-10: DJ42, DJ43, DJ44), alcoholic-related disease or redeemed a prescription with disulfiram (as a crude marker for alcohol abuse) (ICD-8: 291.39, 303.09, 303.19, 303.20, 303.28, 303.29, 571.10, 577.10; ICD-10: DF10, DK70- exclude ICD-10 DK703A, ATC-kode: N07BB01).

In the regression model, we classified cases and controls into the following age categories, ≤50 years, 51–60 years, 61–70 years, 71–80 years or ≥81 years to adjust for potential confounding by age. In the subgroup analyses, we defined the age categories as ≤64, 65–79, and ≥80. The follow-up was divided into two periods, the first from 1 January 2000 to 31 December 2004, and the second periods from 1 January 2005 to 31 December 2009.

We performed subgroup analysis of subjects defined by age, gender, BMI, smoking, alcohol use, glibenclamide use, and gliclazid use. Finally, in order to appraise the duration- and dose–response relationships, we performed some analyses using different cutoff values for treatment duration and cumulative dose.

All analyses were performed using Stata Release 12.0 (StataCorp, College Station, TX).

## Results

**Figure 1:**
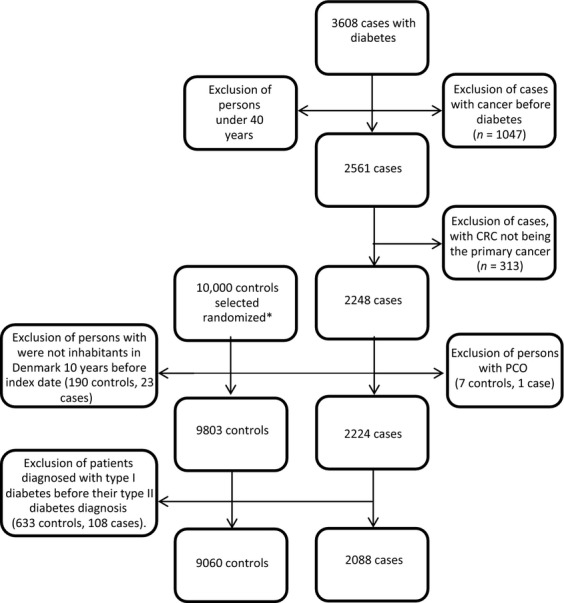
Flowchart: Inclusions- and exclusions of cases and controls. *Controls selected by the same criteria as the cases. Some of these exclusions were performed after the control selection procedure as shown at the flowchart.

In the study period, a total of 3608 CRCs were registered among type II diabetics. We excluded 1520 cases due to our exclusion criteria. The final study population consisted of 2088 cases and 9060 controls rendering an average of 4.3 controls per case (Fig.1). The characteristics of cases and controls are detailed in Table 1.

**Table 1:** Characteristics of cases and controls

	Cases (*n* = 2088)	Controls (*n* = 9060)
Gender		
Men	1268 (60.7%)	4859 (53.6%)
Women	820 (39.3%)	4201 (46.4%)
Age (IQR)	73 (66–80)	69 (61–77)
Metformin. ever user	997 (47.7%)	4448 (49.1%)
Metformin. long-term users	164 (7.9%)	842 (9.3%)
Drug		
All insulins^1^	340 (16.3%)	2146 (23.7%)
Insulin glargine	5 (0.2%)	26 (0.3%)
Glibenclamide	198 (9.5%)	799 (8.8%)
Gliclazide	91 (4.4%)	356 (3.9%)
Rosiglitazone	12 (0.6%)	57 (0.6%)
Asprin	976 (46.7%)	4149 (45.8%)
NSAIDs^2^	149 (7.1%)	856 (9.4%)
Statins	673 (32.2%)	2996 (33.1%)
Diagnosis		
Obesity	332 (15.9%)	1669 (18.4%)
Tobacco use	220 (10.5%)	759 (8.4%)
Alcohol use	114 (5.5%)	565 (6.2%)

1 Including ATC-code A10A. 1 DDD = 40 units for all insulin therapy.

2 Excluding glucosamine.

A subgroup analyses of the association between long-term use metformin use and CRC generated adjusted OR's of 0.83 (95% CI; 0.68–1.00), 0.96 (95% CI; 0.75–1.23) and 0.66 (95% CI; 0.49–0.90) for all subjects, men, and women, respectively (Table 2). Generally, crude OR and adjusted OR differed only slightly.

**Table 2:** Subgroup analysis: association between metformin and CRC in subgroups of patients with given characteristics

	Cases	Controls	Crude OR (95% CI)	Adjusted OR (95% CI)^1^
	Exposed/nonexposed	Exposed/nonexposed		
Total	164/1091	842/4612	0.82 (0.69–0.99)	0.83 (0.68–1.00)
Men	104/653	413/2492	0.96 (0.76–1.21)	0.96 (0.75–1.23)
Woman	60/438	429/2120	0.68 (0.51–0.90)	0.66 (0.49–0.90)
Age <65 year	41/192	318/1565	1.05 (0.73–1.50)	0.82 (0.55–1.22)
Age 65–79 year	91/570	428/1980	0.74 (0.58–0.94)	0.77 (0.59–0.99)
Age >80 year	32/329	96/1067	1.08 (0.71–1.64)	1.06 (0.68–1.63)
Nonconfounding antidibetics^2^	123/985	630/4208	0.83 (0.68–1.02)	0.83 (0.67–1.03)
Marker of obesity	40/122	256/575	0.74 (0.50–1.08)	0.71 (0.47–1.08)
No marker of obesity	124/969	586/4037	0.88 (0.72–1.08)	0.86 (0.69–1.07)
Marker of tobacco use	22/108	64/406	1.29 (0.76–2.19)	1.34 (0.74–2.41)
No marker of tobacco use	142/983	778/4206	0.78 (0.64–0.95)	0.78 (0.63–0.95)
Marker of alcohol use	10/66	32/329	1.56 (0.73–3.32)	1.45 (0.60–3.53)
No marker of alcohol use	154/1025	810/4283	0.79 (0.66–0.96)	0.80 (0.66–0.98)

1 Adjusted for age, gender, calendar year, tobacco, obesity, alcohol use, aspirin, NSAIDs, statins, glibenclamide, and gliclazide.

2 Excluding Glibenclamide and Gliclazide.

The adjusted OR was slightly below unity for subjects with a marker of obesity (OR, 0.71), whereas the adjusted OR was above unity for subjects with a marker of tobacco or alcohol use (OR, 1.56–1.83) (Table 2). A two-sided test for effect modification was statistically significant for gender and for markers of tobacco and alcohol use.

As for dose–response and duration–response relationship, there was an increasing risk reduction of CRC with cumulative doses of metformin >250 DDD and with use of metformin >1 year (Table 3). Test of trend showed a statistically significant trend for both cumulative doses and for duration.

**Table 3:** Dose–response and duration–response association between metformin and colorectal cancer

	Cases	Controls	Crude OR (95% CI)	Adjusted OR (95% CI)^2^
	Exposed/ nonexposed	Exposed/ nonexposed		
Cumulative dose^1^				
DDD<250	309/1091	1279/4612	1.02 (0.89–1.18)	1.09 (0.94–1.26)
DDD 250–499	200/1091	898/4612	0.94 (0.80–1.11)	0.97 (0.81–1.15)
DDD 500–999	324/1091	1429/4612	0.96 (0.84–1.10)	0.98 (0.85–1.13)
DDD 1000–1499	126/1091	630/4612	0.85 (0.69–1.04)	0.86 (0.69–1.06)
DDD ≥1500	38/1091	212/4612	0.76 (0.53–1.08)	0.72 (0.50–1.03)
Duration of use^1^				
<1 year	180/572	627/2147	1.08 (0.89–1.30)	1.09 (0.89–1.32)
1–3 years	198/572	827/2147	0.90 (0.75–1.08)	0.94 (0.78–1.14)
3–5 years	156/572	672/2147	0.87 (0.72–1.06)	0.92 (0.75–1.13)
5–7 years	107/572	519/2147	0.77 (0.62–0.97)	0.82 (0.64–1.04)
7–9 years	67/572	326/2147	0.77 (0.58–1.02)	0.79 (0.59–1.06)
>9 years	41/572	153/2147	1.01 (0.70–1.44)	1.03 (0.71–1.49)

Reference is never-users of metformin. DDD, defined daily dose (2000 mg for metformin).

1 *P*_trend_ < 0.05.

2 Adjusted for gender, age, calendar year, obesity, tobacco, alcohol use, NSAIDs, aspirin, glibenclamide, gliclazide, and statins.

Table 4 shows the characteristics of the control group that were associated with use of metformin. We compared long-term users of metformin with nonusers. Subgroup analysis showed that use of glibenclamide, gliclazide, rosiglitazone, insulin glargine, NSAIDs, and statins was associated with exposure (OR, 1.38–14.39), but use of acetylsalicylic acid and insulins was not associated (OR, 0.98–1.09). The adjusted ORs were <1 for renal failure, diabetic nephropathy, diabetic neuropathy, tobacco, and alcohol use (OR, 0.29–0.79). The analysis associated obesity to the exposed controls (OR, 1.87), while a diagnosis of diabetic retinopathy seemed to be equally distributed in to two groups (OR, 1.02) (Table 4).

**Table 4:** Characteristics of metformin users and nonusers in the control group

	Metformin users (*n* = 842)	Metformin nonusers (*n* = 4612)	Adjusted OR (95% Cl)^2^
Gender			
Men	413 (49.0%)	2492 (54.0%)	0.81 (0.70–0.94)
Women	429 (51.0%)	2120 (46.0%)	1.00 Ref
Age(IQR)	68 (61–74)	70 (61–79)	
Drugs			
All insulins	227 (27.0%)	1238 (26.8%)	1.09 (0.93–1.29)
Insulin glargine	6 (0.7%)	15 (0.3%)	2.06 (0.81–5.23)
Glibenclamide	118 (14.0%)	271 (5.9%)	2.36 (1.89–2.94)
Gliclazide	54 (6.4%)	120 (2.6%)	1.99 (1.46–2.72)
Rosiglitazone	39 (4.6%)	1 (0.0%)	14.4 (8.0–25.8)
Aspirin	438 (52.0%)	2011 (43.6%)	0.98 (0.84–1.15)
NSAIDs^1^	115 (13.7%)	400 (8.7%)	1.38 (1.10–1.72)
Statins	451 (53.6%)	1156 (25.1%)	1.88 (1.59–2.21)
Diagnosis			
Renal failure	2 (0.2%)	39 (0.8%)	0.32 (0.08–1.34)
Diabetic retinopathy	130 (15.4%)	653 (14.2%)	1.02 (0.83–1.25)
Diabetic neuropathy	7 (0.8%)	76 (1.6%)	0.52 (0.24–1.14)
Diabetic nephropathy	1 (0.1%)	27 (0.6%)	0.29 (0.04–2.14)
Obesity	256 (30.4%)	575 (12.5%)	1.87 (1.58–2.21)
Tobacco	64 (7.6%)	406 (8.8%)	0.79 (0.60–1.05)
Alcohol use	32 (3.8%)	329 (7.1%)	0.60 (0.41–0.87)

1 Excluding aspirin.

2 Adjusted for gender, age, calendar year, obesity, tobacco, alcohol use, NSAIDs, aspirin, glibenclamide, gliclazide og statins.

## Discussion

Our study showed a protective effect of metformin on the risk of developing CRC with an adjusted OR of 0.83 (95% CI 0.68–1.00). A surprising finding was a strong interaction by gender, which was not an a priori defined hypothesis and thus it needs corroboration from other studies before any firm inferences can be made. Finally, we were able to demonstrate a clear dose–response and duration–response effect.

### Comparison with previous studies

In recent years, epidemiological evidence has indicated that metformin possibly prevents CRC 15,24–29. A meta-analysis of 13 studies, 12 observational and 1 randomized study assessed the association between metformin and CRC. When analyzing the observational studies, it showed that the risk of CRC was decreased by 17% (OR 0.83, 95% CI 0.74–0.92) among patients treated with metformin compared to those not using metformin. This supported our finding (OR 8.83, 95% CI 0.68–1.00). However, this association was not seen in the randomized trial (OR 1.02, 95% CI 0.41–2.5). Since many of the observational studies in the meta-analysis were not designed to evaluate the effect of metformin on the outcome CRC, Franciosi et al. concluded that intervention studies designed to evaluate this were required.

Several of the observational studies included in the meta-analysis evaluated the association of metformin and cancer as a secondary analysis 27,28. They sampled from different registries as The Longitudinal Health Insurance Database 2000, the General Practice Research Database, The Health Information Network, and the Health Informatics Centre 27,29–31. The population in Lee et al. was noncaucasians and was therefore not directly comparable to our study population 29. Morden et al. only included diabetics >68 years 28. In Lee et al., the metformin use was 500 mg/day, which extrapolated to 5 years would be approximately 450 DDD, compared to the 1000 DDD in our study 29. None of the studies excluded individuals with IBD, which is a potential risk factor of CRC 32,33. Ruiter et al. were not able to identify and exclude patients who used metformin for other indications, for example, PCOS 34. Aside from the study by Lee et al., ours is the first address metformin use versus no metformin use with CRC as a predefined primary outcome among type II diabetics.

### Gender interaction

There was a strong interaction by gender. In women, there was a protective effect of long-term metformin use against CRC (OR = 0.66, 95% CI 0.49 – 0.90), which was not seen in men (OR = 0.96, 95% CI 0.75 – 1.23). This was not a predefined hypothesis. We were not aware of any gender-specific pharmacological action of metformin or CRC tumor biology that could explain this difference. In light of the recent finding of pronounced gender differences in the cardioprotective effect of metformin, we did not find it unlikely that similar gender differences could occur for the cancer-preventive effect 35. Furthermore, several other studies have reported a moderate gender difference in the metformin–cancer association, without commenting on it 30,31.

### The association between dose–response and duration–response of metformin use and CRC

The study showed a statistical significant dose–response association between the use of metformin and a reduced risk of CRC. The protective effect of metformin ceased after 9 years of treatment (OR 1.03). Our dose–response analysis supported the dose–response association found by previous studies 4,34. However, this is the first time a duration–response association is found.

### Comparison of metformin users and nonusers in the control group

To address the potential for confounding and to confirm or disconfirm the associations identified by Tzoulaki et al., we analyzed the association between different covariates and metformin exposure among the controls. Tzoulaki et al. identified that metformin users had a higher BMI, lower age, lower systolic blood pressure, a lower prevalence of cardiac diseases, and a greater use of Aspirin and NSAIDs compared to users of SU-drugs 36. Our controls are representative of the covariate distribution in the source population, that is, type 2 diabetics. With the exception of use of rosiglitazone (OR = 14.4) and renal failure (OR = 0.32), all associations were weak. Both of the strong associations were expected; rosiglitazone was marketed as a combination drug with metformin, and metformin is usually avoided in renal failure due to the risk of lactic acidosis. To our knowledge, there is no known association between cancer and either rosiglitazone use or renal failure. The fact that all other characteristics were weakly associated with metformin use limits the potential for these variables to act as confounders.

### Strength and weaknesses of the study

Among the strengths of our study, we only included cases with histologically verified cancers. We restricted our study population to subjects with a long and stable residency and to only consist of type II diabetics. The latter was made by using NPR which is another strength of the study. There is an association between type II diabetes and cancer development, and both diseases share many of the same sociodemographic and life style risks, for example, age, gender, BMI, physical activity that play a critical role in the progression of the two diseases 37, 38.

We could not include type II diabetics that only have been in contact with the primary health care system. However, this proportion is likely to be small. Due to diabetes-related comorbidity, the patients are often both in the primary and secondary health care system. In addition, we have no reason to think that the metformin–CRC association would be different in diabetics treated only in primary care. Another limitation of our study is the lack of data on lifestyle factors that might confound the metformin–CRC association. Obesity, tobacco smoke, and alcohol use are all associated with a greater risk of CRC 39–42, and thus have the potential to confound our results. We stratified our data using crude proxies of these lifestyle factors (Table 2). There was no indication of important confounding, but a moderate effect modification by markers of alcohol and tobacco use. Table 4 indicates a weak inverse association between alcohol and tobacco and metformin use. Given that our markers all have limited sensitivity, we cannot rule out that some of the protective effect might be explained by residual confounding by alcohol and smoking. On the other hand, there is a clear positive association between obesity and metformin use (OR = 1.87). Our study population had 18% subjects with an obesity diagnosis, whereas the literature indicated that about 80% of all type II diabetics are visceral obese 43,30. Thus, we might have some residual confounding by incomplete registering of truly obese subjects. In contrast to the potential alcohol and smoking confounding, obesity would work in the opposite direction. Obesity is a risk factor for CRC and is linked to metformin use. However, this confounder has the direction of elevating the OR. Thus, implying that when we observe an apparent protective effect, this is still valid. We may have underestimated the protective effect, though.

To our knowledge, there is no strong association between renal failure and CRC, and therefore we did not include renal failure as a potential confounder.

What are the consequences of a CRC preventive effect of metformin? At least, there seems to be a collateral benefit in persons who have other indications for metformin. However, given the potential adverse effects of its use, metformin would be an inappropriate agent for large-scale chemoprevention in healthy subjects. The next step would be to identify the underlying molecular basis for its anticancer effect, which might eventually lead to the development of other drugs with a more specific chemopreventive effect.
